# Supplementary material for: Gas Chromatography–Mass Spectrometry-Based Cerebrospinal Fluid Metabolomics to Reveal the Protection of Coptisine against Transient Focal Cerebral Ischemia–Reperfusion Injury via Anti-Inflammation and Antioxidant
Source: Molecules. 2023 Aug 30;28(17):6350. doi: 10.3390/molecules28176350 (PMC10489949; doi:10.3390/molecules28176350)
Supplement: Supplementary file 1 [file molecules-28-06350-s001.zip › molecules-2565136-supplementary.pdf]

Table S1. Retention time and similarity of analytes in SD rats' CSF by GC-MS

Compound	tR（min）	Similarity (%)	Compound	tR（min）	Similarity (%)
Cyclobutane	2.027	80	Pentanedioic acid	17.315	88
3-Buten-2-ol	2.415	81	Glutamine	17.904	92
alpha-Pinene	3.185	96	Phenylalanine	17.959	95
Ethane	3.225	97	Ribonic acid	18.205	80
2-Thenaldehyde	3.38	81	Dodecanoic acid	18.345	83
Benzene	3.435	86	Lyxose	18.37	86
borate	3.605	93	Ribose	18.4	92
ethane-1,2-diol	3.67	92	Asparagine	18.521	93
Dimethylamine	4.05	93	Arabinose	18.579	89
Pentane	4.655	95	Lysine	18.715	81
Propanoic acid	4.775	93	Sorbitol	18.805	89
Acetic acid	5.315	93	Arabitol	19.035	90
n-Butylamine	5.705	92	Phosphoric acid	19.482	87
Alanine	5.885	92	Galactopyranose	19.94	96
1,4-Butanediol	5.92	84	1,2,3-PA	20.03	86
Butanoic acid	6.415	94	D-Ribo-Hexitol	20.075	85
1,2-Butanediol	6.73	83	Gluconic acid	20.11	83
Ethanedioic acid	6.78	82	Erythrotetrofuranose	20.325	84
Valine	8.628	88	Fructose	20.47	93
m-ethynylaniline	8.7	84	Galactose	20.595	93
Urea	9.415	93	Glucose	20.69	89
Glycerol	10.185	93	Dulcitol	21.01	91
2(3H)-Furanone	10.25	92	Ribitol	21.011	84
Isoleucine	10.676	93	Tyrosine	21.085	87
Threonine	10.695	90	Sucrose	21.18	85
Glycine	10.98	94	Cholesterol	21.266	86
Butanedioic acid	11.295	94	Pantothenic acid	21.45	82
Chlorphentermine	11.373	80	Mannose	21.639	83
2,3-DBA	11.992	91	Palmitelaidic acid	21.711	83
Serine	12.48	92	Hexadecanoic acid	21.825	91
Cadaverine	13.753	84	Inositol	22.026	85
AA	15.322	83	Uric acid	22.079	84
Malic acid	15.762	90	Stearic acid	22.795	84
Threitol	16.13	90	Oleic acid	22.93	89
Proline	16.33	96	2-monopalmitin	23.35	84
Aspartic acid	16.376	90	2-Monostearin	23.745	86
Pentanoic acid	16.545	82	13-Docosenamide	24.195	89
Creatinine	16.87	91	1,2-BA	24.813	86
2,3,4-TBA	17.03	95			

Abbreviation：1,2,3-PA:1,2,3-Propanetricarboxylic acid;2,3,4-TBA:2,3,4-Trihydroxybutyric acid；1,2-BA: 1,2-Benzenedicarboxylic acid; 2,3-DBA:2,3-Dihydroxybutanoic acid;

Table S2. Reproducibility of analytes in Quality Control from CSF by GC-MS

Metabolites	tR(min)			Content（ng/mL)		
	Mean	SD	RSD(%)	Mean	SD	RSD(%)
Alanine	5.889	0.002	0.037	0.158	0.009	5.873
Serine	12.489	0.005	0.040	0.237	0.007	2.853
Cadaverine	13.754	0.005	0.037	0.058	0.002	4.139
Glucose	20.692	0.003	0.013	10.506	0.475	4.523
Sucrose	21.182	0.003	0.014	0.195	0.007	3.578
Stearic acid	22.794	0.004	0.016	0.063	0.002	2.584

Abbreviation:RSD: relative standard deviation.SD:standard deviation.

Table S3. Screen in CSF marker between control and cerebral IR

Compound	Log2(FC)	Log10(p)	Compound	Log2(FC)	Log10(p)
Lysine	-5.432	2.703	Arabitol	-1.890	5.254
Propanoic acid	-5.066	9.415	2(3H)-Furanone	-1.737	1.302
13-Docosenamide	-3.794	5.836	Dodecanoic acid	-1.338	2.119
Sucrose	-3.222	5.474	n-Butylamine	-1.288	1.543
Gluconic acid	-3.100	2.913	Acetic acid	-0.821	4.083
2,3,4-TBA	-3.036	7.270	1,2,3-PA	-0.811	2.231
2-monopalmitin	-2.887	7.536	Hexadecanoic acid	-0.712	2.109
D-Fructose	-2.881	6.850	ethane-1,2-diol	0.831	2.794
Dulcitol	-2.782	8.923	Pantothenic acid	0.942	1.452
Threitol	-2.717	6.489	Ethanedioic acid	1.161	1.905
Lyxose	-2.614	11.446	Glucose	1.650	7.664
2-Monostearin	-2.573	1.932	2-Thenaldehyde	2.490	3.221
Ribonic acid	-2.561	11.687	Alanine	2.689	3.122
Glycerol	-2.540	9.927	borate	2.698	10.277
Galactopyranose	-2.540	1.811	Tyrosine	2.744	8.730
Ethane	-2.514	13.378	Ribose	2.914	8.069
Cadaverine	-2.490	1.939	Erythrotetrofuranose	3.032	7.199
Dimethylamine	-2.421	13.737	Butanoic acid	3.193	0.656
3-Buten-2-ol	-2.390	15.900	Creatinine	3.555	6.126
Sorbitol	-2.323	3.251	Serine	3.577	6.291
Oleic acid	-2.309	1.387	Pentane	3.772	6.677
D-Ribo-Hexitol	-2.050	1.723	1,4-Butanediol	4.049	6.168
Glycine	-2.007	3.464	Galactose	4.068	1.691
1,2-Butanediol	-1.957	1.993			

Table S4. The candidate markers of CSF between cerebral IR and Cop treated.

Compound	Log2(FC)	-Log10(p)
Ribose	-2.502	4.533
Stearic acid	-2.432	1.830
Glycerol	2.742	3.095
2,3,4-TBA	0.587	1.435
Glycine	2.852	1.834
Oleic acid	4.170	4.674

Table S5. Identification in CSF markers between control, cerebral IR and Cop treated groups

Compounds	Control vs Cerebral IR		Cerebral IR vs Cop treated	
	Log2(FC)	-Log10(p)	Log2(FC)	-Log10(p)
Ribose	2.914	8.069	-2.502	4.533
Glycerol	-2.540	9.927	2.742	3.095
2,3,4-TBA	-3.036	7.270	0.587	1.435
Glycine	-2.007	3.464	2.852	1.834
Oleic acid	-2.309	1.387	4.170	4.674
